# Intracellular cholesterol transport inhibition Impairs autophagy flux by decreasing autophagosome–lysosome fusion

**DOI:** 10.1186/s12964-022-00942-z

**Published:** 2022-11-25

**Authors:** Yunash Maharjan, Raghbendra Kumar Dutta, Jinbae Son, Xiaofan Wei, Channy Park, Hyug Moo Kwon, Raekil Park

**Affiliations:** 1 Department of Biomedical Science and Engineering, Gwangju Institute of Science and Technology, Gwangju, 61005 Republic of Korea; 2 School of Life Sciences, Ulsan National Institute of Science and Technology, Ulsan, Republic of Korea; 3 School of Dentistry, Philips Institute for Oral Health Research, Virginia Commonwealth University, Richmond, VA USA; 4 Present Address: Department of Oral and Craniofacial Molecular Biology, School of Dentistry, Virginia Commonwealth University, Richmond, VA USA

**Keywords:** Intracellular cholesterol transport, Autophagosomes, Autophagy flux, Chloroquine, U18666A, Bafilomycin A1, STX17

## Abstract

**Background:**

Autophagy is an intracellular degradation process crucial for homeostasis. During autophagy, a double-membrane autophagosome fuses with lysosome through SNARE machinery STX17 to form autolysosome for degradation of damaged organelle. Whereas defective autophagy enhances cholesterol accumulation in the lysosome and impaired autophagic flux that results Niemann-Pick type C1 (NPC1) disease. However, exact interconnection between NPC1 and autophagic flux remain obscure due to the existence of controversial reports.

**Results:**

This study aimed at a comparison of the effects of three autophagic inhibitor drugs, including chloroquine, U18666A, and bafilomycin A1, on the intracellular cholesterol transport and autophagy flux. Chloroquine, an autophagic flux inhibitor; U1866A, a NPC1 inhibitor, and bafilomycin A, a lysosomotropic agent are well known to inhibit autophagy by different mechanism. Here we showed that treatment with U1866A and bafilomycin A induces lysosomal cholesterol accumulation that prevented autophagic flux by decreasing autophagosome–lysosome fusion. We also demonstrated that accumulation of cholesterol within the lysosome did not affect lysosomal pH. Although the clearance of accumulated cholesterol by cyclodextrin restored the defective autophagosome–lysosome fusion, the autophagy flux restoration was possible only when lysosomal acidification was not altered. In addition, a failure of STX17 trafficking to autophagosomes plays a key role in prevention of autophagy flux caused by intracellular cholesterol transport inhibitors.

**Conclusions:**

Our data provide a new insight that the impaired autophagy flux does not necessarily result in lysosomal cholesterol accumulation even though it prevents autophagosome–lysosome fusion.

**Video abstract**

**Supplementary Information:**

The online version contains supplementary material available at 10.1186/s12964-022-00942-z.

## Introduction

Autophagy is a degradation process eliminating aggregated proteins and damaged organelles to maintain cellular homeostasis (1, 2). Once activated, autophagy continuously degrades cellular component through autophagy flux and encompasses the formation of autophagosome whose fusion with lysosome form autolysosome where enzymatic degradation happens (3, 4). Autolysosome formation is tightly regulated by soluble N-ethylmaleimide-sensitive factor attachment protein receptor (SNARE) proteins, including syntaxin 17 (STX17), synaptosomal-associated protein 29 (SNAP29), Rab GTPases, and other tethering complexes (5, 6).

Cellular cholesterol transport is a dynamic process in which LDL cholesterol from the plasma membrane is trafficked into the lysosomes, where it is cleaved by acidic lipase, generating free cholesterol, then transported out of the lysosomes through the action of Niemann Pick type C (NPC) proteins (7). Defect associated with the egress of unesterified cholesterol from the lysosomes result in cholesterol accumulation within the lysosome detected by a fluorescent probe filipin specifically binding to unesterified cholesterol, and not to esterified sterols (7, 8). Studies have reported that the impaired autophagy flux is closely related to lysosomal cholesterol accumulation in Niemann-Pick type C1 (NPC1) disease without any pathological features of anomalous lysosomal proteolytic activities (9). Maturation process of autophagosomes was impaired because of the formation of defective amphisome resulted through a failure in SNARE trafficking (9). Additionally, the clearance of accumulated cholesterol from the lysosome by methyl-β-cyclodextrin enhances autophagosome–lysosome fusion by facilitating interaction among SNARE proteins, thereby recovering autophagic flux in NPC1 deficient cells (10). Interestingly, inhibition of autophagy flux due to the genetic ablation of essential autophagy-related genes tends to show controversial result on lysosomal cholesterol accumulation (9, 11, 12). It is reasonable to speculate that mutual or reciprocal relationship between impaired intracellular cholesterol transport and impaired autophagy.

Pharmacological inhibitors, such as bafilomycin A1 (Vacuolar H^+^ ATPase, V-ATPase inhibitor), chloroquine (lysosomotropic agent), and NH_4_Cl (a weak base), of autophagy are known to prevent lysosomal acidification which results in extensive filipin-cholesterol staining of the lysosome (13), indicating that maintenance of the acidic pH of the lysosome might be essential for cholesterol efflux from the lysosome to other cellular compartment. Although these speculation seem to be reasonable, they contradict NPC1 disease phenotype, which do not show any phenotypic alteration associated with lysosomal acidification. Therefore, the mechanism underlying relationship between lysosomal cholesterol accumulation and lysosomal acidification remains obscure. Thus, lysosomal cholesterol accumulation induced by late-stage autophagy inhibitor could be the outcome of either impaired autophagy flux or prevented autophagy flux. However, it is unknown whether autophagy inhibitor, such as chloroquine and bafilomycin A1, impair autophagy flux by either well-known inherent properties or impact on the autophagy process by stimulating lysosomal cholesterol accumulation. Although U18666A, an inhibitor of intracellular cholesterol transport, obstructs autophagy flux, its underlying mechanism of autophagy inhibition remains unclear (14, 15). Mauthe et al. suggest that chloroquine inhibits autophagic flux by decreasing autophagosome–lysosome fusion without affecting lysosomal acidity, contradicting the established property of chloroquine as a lysosomotropic agent (16). Except for the property of bafilomycin A1 in preventing lysosomal acidification, its role to inhibit the autophagosome–lysosome fusion is questionable (17). Moreover, a rationale of observation that bafilomycin A1 solely attributes to inhibit either V-ATPase (18) or sarco/endoplasmic reticulum Ca^2+^-ATPase (SERCA) (19). Although the exact role of cholesterol on autophagy impairment is unknown, it is critically important to elucidate the impact of lysosomal cholesterol accumulation on autophagy in numerous studies of autophagy inhibitors. It would be more widely beneficial because these inhibitor are frequently used to compare the effect of other potential therapeutic drug targeting autophagy.

This study compared the effect of autophagy inhibitors, including chloroquine, bafilomycin A1 and U18666A, on autophagy flux and cholesterol accumulation. The results demonstrated that lysosomal cholesterol accumulation impaired autophagy flux by preventing autophagosome–lysosome fusion. However, impaired autophagy flux did not necessarily result in lysosomal cholesterol accumulation.

## Materials and methods

### Materials

#### Reagent

Chloroquine (#C6628, Sigma-Aldrich), U18666A (#1638, Tocris), bafilomycin A1 (#1793, Sigma-Aldrich), filipin (#4767, Sigma-Aldrich), Lysotracker Red (#L7528, Invitrogen /Thermo Fisher Scientific), methyl-β-cyclodextrin (#C4555, Sigma-Aldrich), and thapsigargin (#T9033, Sigma-Aldrich).

#### Antibodies

LC3 (#L8918, Sigma-Aldrich), SQSTM1/p62 (#H00008878-M01, Abnova), ACTB (#sc-47778, Santa Cruz), STX17 (#GTX130212, GeneTex), LAMP1 (#L1418, Sigma-Aldrich), and ATG7 (#2631S, Cell Signalling Technology).

### Cell culture

Retinal pigment epithelial-1 (RPE1), RPE1-RFP-GFP-LC3, and mouse embryonic fibroblasts (MEFs) were cultured in a high-glucose Dulbecco’s modified Eagle medium (DMEM; #11,965,092, Gibco), supplemented with 10% fetal bovine serum (FBS; #16,000,044, Gibco), and 100 IU/mL penicillin and 100 µg/mL streptomycin (#15,070,063, Gibco), at 37 °C and 5% CO_2_ in a humid atmosphere. All cells were confirmed to be negative for mycoplasma contamination.

### Generation of htRPE1-mRFP-GFP-LC3

RPE1 cells were transfected with 2 μg of RFP-GFP-LC3 plasmids using X-tremeGENE HP DNA transfection reagent (Sigma-Aldrich, 6,366,244,001) to generate stable cell lines, expressing ptfLC3 (#21,074, Addgene; deposited by Tamotsu Yoshimori). Cells were switched to a medium supplemented with 700 mg/mL G418 (#A1720, Sigma-Aldrich) at 24 h after transfection to select the neomycin-resistant cells. Fresh medium was replaced every 2–3 days until colonies were formed at ~ 15 days. Individual colonies were isolated using cloning cylinders (#C1059, Merck) and a fluorescence microscope ((IX73, Olympus) was used to assess the expression of RFP-GFP-LC3.

### GFP-LC3 plasmid transfection

Following the manufacturer’s transfection protocol, green fluorescent protein-LC3 (GFP-LC3) (1 μg/ml of culture media) was transfected using Lipofectamine 3000 reagent (#L3000015, Invitrogen).

### Immunofluorescence

Cells grown on coverslip were rinsed thrice with 1X phosphate buffered saline (PBS, pH 7.4) and fixed with 4% paraformaldehyde for 30 min at room temperature. They were then rinsed thrice with PBS, permeabilized with 0.25% Triton X-100 for 10 min, again rinsed thrice with PBS, and blocked with 3% bovine serum albumin (BSA) for 1 h at room temperature. Cells were then incubated with primary antibodies in 3% BSA, rinsed thrice with PBS, and labeled with fluorescent Alexa Fluor 568 (molecular probes)-conjugated secondary antibodies (1:500) for 30 min. Coverslips were mounted on slide with Prolong Gold antifade reagent containing DAPI (#P36931, Sigma) and examined under an Olympus Fluoview 1000 confocal laser-scanning microscope or a fluorescence microscope (IX71, Olympus).

### Quantification of fluorescent colocalization

Fluorescence intensity within co-immunostained cells was measured using ImageJ software. Co-localisation was measured using colocalization plugin by calculating Manders’ overlap coefficients (OC), which quantifies overlapping pixels from each channel. The OC of lysosome fluorescent signal and GFP-LC3 fluorescent signal were quantitatively assessed to compute the percentage of the lysosomes overlapping with GFP-LC3. The OC value of 1 was defined as 100% and each OC was expressed relative to 100% value. The percentage of the lysosomes overlapping with GFP-LC3 ranging between that in the control and the experimental groups was interpreted as the fold changes over the control.

### Small interfering RNA (siRNA) transfection

Cells were transfected with a combination of Atg7 (#1 CUGUUCACCCAAAGUUCUU [#1,325,032, Bioneer] and #2 CAGACAAGAAGCUCCUUCU [#1,325,027, Bioneer]) and scrambled control siRNAs (#sc-37007, Santa Cruz Biotechnology), using Lipofectamine RNAiMAX (#13,778–150, Invitrogen) according to the manufacturer’s protocol.

### RNA extraction and reverse transcription-qPCR

Total RNA was extracted from the samples as described previously (20, 21). A reverse transcription kit (Roche, Indianapolis, IN, USA) was used to transcribe cDNA. qPCR was performed with cDNA as a template using a light cycler system with FastStart SYBR Green Master (#4,673,492,001, Roche). Human primer sequences (forward and reverse) were as follows: intracellular control *36B4*, 5′-TGCATCAGTACCCCATTCTATCA-3′, 5′-AAGGTGTAATCCGTCTCCACAGA 3′; *HMG-CoA synthase*, 5′-GACTTGTGCATTCAAACATAGCAA-3′, 5′-GCTGTAGCAGGGAGTCTTGGTACT -3′; *HMGCoA reductase*, 5′- CAAGGAGCATGCAAAGATAATCC-3′, 5′-GCCATTACGGTCCCACAC -3′; *INSIG1*, 5′- CCCAGATTTCCTCTATATTCGTTCTT-3′, 5′-CACCCATAGCTAACTGTCGTCCTA-3′; *CHOP*, 5′- CAGAGCTGGAACCTGAGGAG-3′, 5′-TGGATCAGTCTGGAAAAGCA -3′; *TRB3*, 5′-GTCTTCGCTGACCGTGAGA -3′, 5′-CAGTCAGCACGCAGGAGT -3′.

### Filipin staining and intensity acquisition

Filipin staining and intensity acquisition were performed as previously reported (22). Cells grown on a coverslip were fixed with 4% paraformaldehyde in PBS for 30 min at room temperature and rinsed thrice with PBS. Paraformaldehyde was quenched with 1.5 mg/mL glycine in PBS for 10 min. Subsequently, 25 μg/mL of filipin in PBS was added, incubated for 2 h at room temperature, and rinsed thrice with PBS. Coverslips were mounted on slides using 90% (v/v) glycerol. Images were acquired using fluorescence microscope (IX71, Olympus), setting the same fixed exposure time for all the samples in each experiment. ImageJ software was used to analyze the images and measure the mean fluorescence intensity in the region of interest based on cell shape. Filipin intensity data were interpreted as arbitrary units, as shown by ImageJ software.

### Lysotracker staining and intensity acquisition

Lysotracker staining was performed according to the manufacturer’s instructions. Images were acquired using a fluorescence microscope (IX71, Olympus), setting the same fixed exposure time for all the samples in each experiment. ImageJ software was used to quantify the images and measure the mean fluorescence intensity in the region of interest, based on cell shape. Difference in lysotracker intensity between the control and the test groups was interpreted as the fold changes over the control.

### Whole-cell lysates

Cells were lysed on ice in radioimmunoprecipitation assay (RIPA) lysis buffer (20 mM HEPES pH 7.5, 150 mM NaCl, 1% Triton X-100, 1% sodium deoxycholate, 1 mM EDTA), supplemented with protease and phosphatase inhibitors (#PPC1010, Sigma-Aldrich), and centrifuged at 14,000 rpm for 10 min at 4 °C. Sodium dodecyl sulfate (SDS) loading buffer was added to the supernatant and denatured at 95 °C for 5 min.

### Western blotting

Aliquots of each protein lysate were subjected to SDS-PAGE for 120 min at 100 V. Proteins were then transferred to nitrocellulose membranes for 120 min at 300 mA and blocked for 30 min with 5% skim milk in Tris-buffered saline with Tween buffer (TBST). Membranes were incubated with primary antibodies overnight at 4 °C and then incubated with peroxidase-coupled secondary antibodies for 1 h at room temperature. Antibody-targeted proteins were visualized using Western blot detection kit (#LF-QC0103, Abfrontier). The visuals from Western blot were measured using ImageJ software, and data were interpreted as the fold changes over the control.

## Result

### U18666A and chloroquine differentially inhibit autophagy flux

This study sought to determine whether autophagy impairment resulted by pharmacological inhibitor of autophagy flux, such as chloroquine, causes lysosomal cholesterol accumulation. It also questioned whether pharmacological inhibition of intracellular cholesterol transport by U18666A contributes to autophagy impairment. First, the optimal concentrations of chloroquine and U18666A, sufficient to induce maximum autophagosome accumulation and intracellular cholesterol transport over a longer incubation period in a serum-starved condition, were determined. This strategy allowed us to determine whether lysosomal cholesterol accumulation governs autophagy impairment or vice versa when each process in question is analyzed in a time course followed by correlation analysis. Furthermore, serum-starved condition from the time of drug treatment ensure that only cholesterol present within cell would be responsible for its effect on cellular physiology if pharmacological agents tend to control intracellular cholesterol homeostasis.

**Fig. 1:**
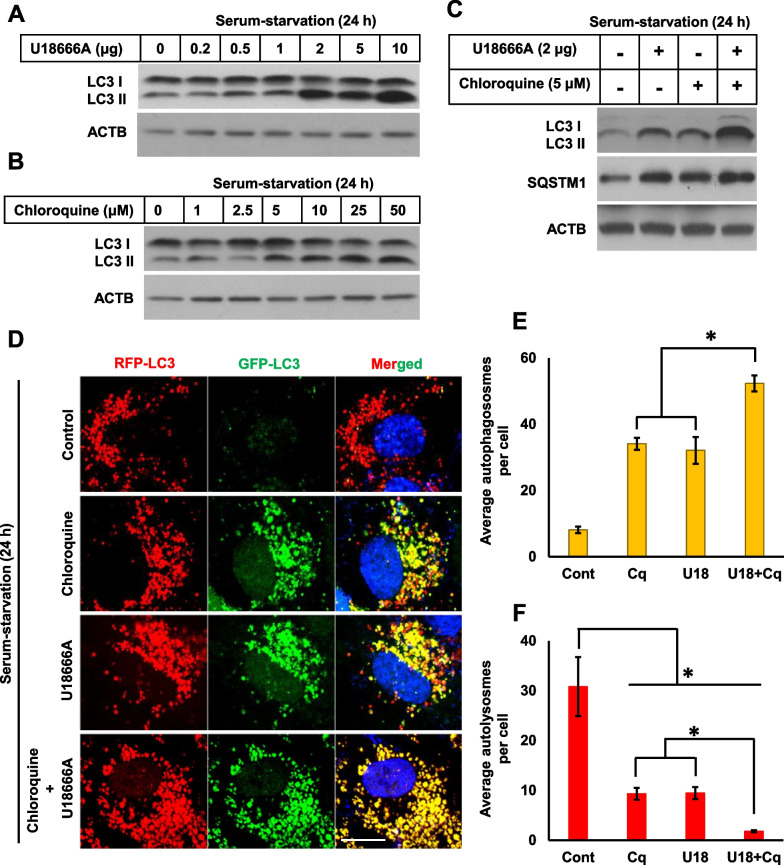
U18666A and chloroquine impair autophagy through different mechanisms. **A** RPE1 cells treated with different concentrations of U18666A in a serum-starvation medium for 24 h. Cells were then subjected to Western blot for LC3 and ACTB. **B** Cells treated with different concentrations of chloroquine in a serum-starvation medium for 24 h. Cells were then subjected to Western blot for LC3 and ACTB. **C** Cells were maintained with U18666A (2 μg), chloroquine (5 μM), or combined treatment with both drugs in a serum-starvation for 24 h. Cells were then subjected to Western blot for LC3, SQSTM1, and ACTB. **D** RPE1-mRFP-GFP-LC3 cells treated as in **C**, fixed with 4% paraformaldehyde, and stained with DAPI. Scale bar, 10 μm. **E, F**, Autophagy status is determined by counting autophagosomes (yellow puncta with both GFP and RFP signal) or autolysosomes (punctate structures with only RFP signal). At least 100 cells were counted for each experimental group. The bar graph represents the mean ± SD (n = 3 experiments). *P < 0.05, Student's t-test

Figure 1A showed a drastic increase in LC3-II protein expression, suggesting that treatment of RPE1 cells with 2 μg/ml of U18666A for 24 h was sufficient to induce autophagosome accumulation. Eventhough an increase in LC3-II expression depended upon concentration, no observable difference was recorded in LC3-II accumulation over 2 μg/ml of U18666A. Treatment with 5 μM chloroquine was sufficient to induce LC3-II accumulation. Similarly, no distinctive difference was shown in LC3-II accumulation after treatment over 5 μM of chloroquine (Fig. 1B), suggesting that autophagosome accumulation tends to be saturated within a certain concentration range of U18666A and chloroquine. Following experiments were done with 2 μg/ml U18666A and 5 μM chloroquine. Combined treatment was employed to test whether these two compounds inhibited autophagy flux through either the same or different mechanisms. Interestingly, these result highlighted that combined treatment with U18666A and chloroquine markedly increased LC3-II accumulation compared to that observed using each compound alone, suggesting different mechanism of the two drugs in inhibition of autophagy flux were evidenced (Fig. 1C). To further confirm that U18666A and chloroquine inhibit autophagy flux, RPE1 cells, stably expressing mRFP-GFP-LC3, were used to measure autophagosome accumulation. LC3 puncta, containing both GFP and RFP signals, represented autophagosomes (shown as yellow fluorescence), whereas LC3 puncta with only RFP signals represent autolysosomes (shown as red fluorescence) as GFP signal is quenched by lysosomal pH (14). Both U18666A and chloroquine significantly induced the autophagosome accumulation to a comparable level, and the combination of two compounds further increased autophagosomes and profoundly reduced autolysosomes (Fig. 1D–F). Taken together, these data suggest that U18666A and chloroquine resulted in autophagy impairment through a negative regulation of autolysosome formation. However, the two drugs are different in detail of mechanisms.

### Lysosomal cholesterol sequestration induced by U18666A significantly correlates with impairment of autophagy flux

**Fig. 2:**
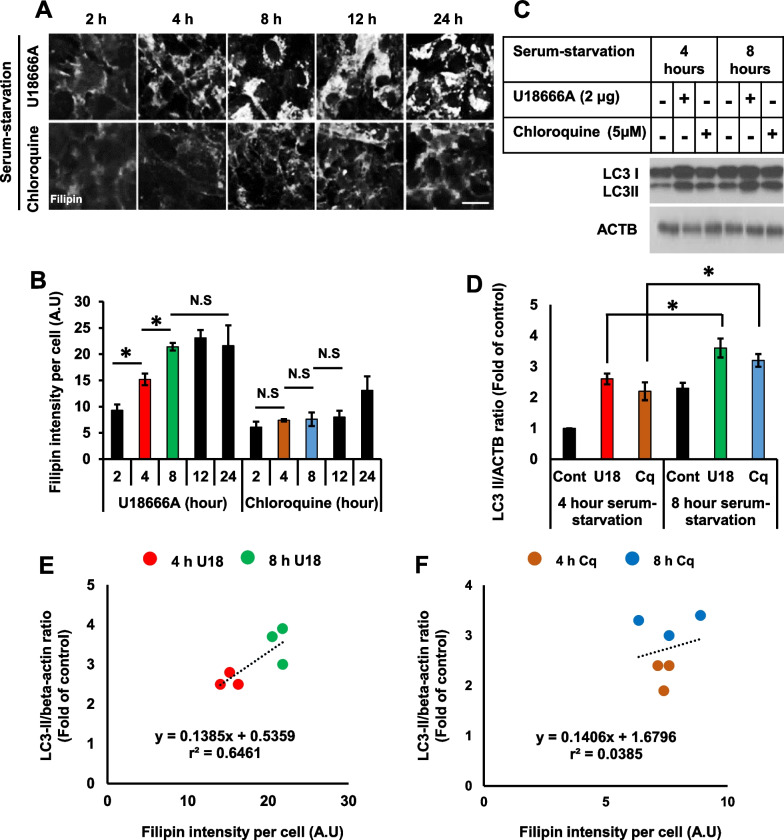
Autophagy impairment by U18666A significantly correlates with lysosomal cholesterol sequestration. **A** RPE1 cells were treated with either U18666A (2 μg) or chloroquine (5 μM) in a serum-starvation medium for different durations. Cells were then subjected to filipin staining. Scale bar, 10 μm. **B** Fluorescence intensity of filipin was measured using ImageJ software. Filipin intensity was compared for different time points in the presence of U18666A (2 μg) or chloroquine (5 μM). Filipin intensity from 50 cells was acquired for each experimental group. The bar graph represents the mean ± SD (n = 3 experiments). *P < 0.05, Student's t-test. **C** Cells treated with either U18666A (2 μg) or chloroquine (5 μM) in a serum-starvation medium for 4 h or 8 h. Cells were then subjected to Western blot for LC3 and ACTB. **D** Intensity of the bands from **C** was quantified using Image J software, and the LC3-II/ACTB ratio was measured. Bar graph represents the mean ± SD (n = 3 experiments). *P < 0.05, Student's t-test. **E, F** Pearson’s correlation coefficient for the association between the impaired autophagy flux and cholesterol accumulation between 4 and 8 h in the presence of U18666A or chloroquine was determined by plotting individual LC3-II/ACTB ratio and filipin intensity from three different experiments

Time-course studies of filipin staining and LC3-II accumulation were performed to determine the relationship between lysosomal cholesterol accumulation and impaired autophagy flux. The enhanced filipin intensity observed as early as at 4 h and saturated at 8 h, suggesting that treatment with U18666A (2 μg/ml) markedly resulted in cholesterol accumulation in a time-dependent manner (Fig. 2A, B). Contrastingly, treatment with chloroquine (5 μM) did not enhance the remarkable intensity of filipin for 24 h. Both U18666A and chloroquine accumulated autophagosomes as early as at 4 h, which was further augmented at 8 h (Fig. 2C, D). We employed Pearson’s correlation coefficient to assess the correlation between an increase in autophagosomes (LC3/ACTB ratio, Fig. 2D) and lysosomal cholesterol content (filipin intensity, Fig. 2A) observed between at 4 h and at 8 h after treatment. In cells treated with U18666A, Pearson’s correlation coefficient was significantly correlated, with r^2^ = 0.6461, at 4 h and 8 h (Fig. 2E). However, it was statistically insignificant, with r^2^ = 0.0385 (Fig. 2F), in cells treated with chloroquine at any time points.

**Fig. 3:**
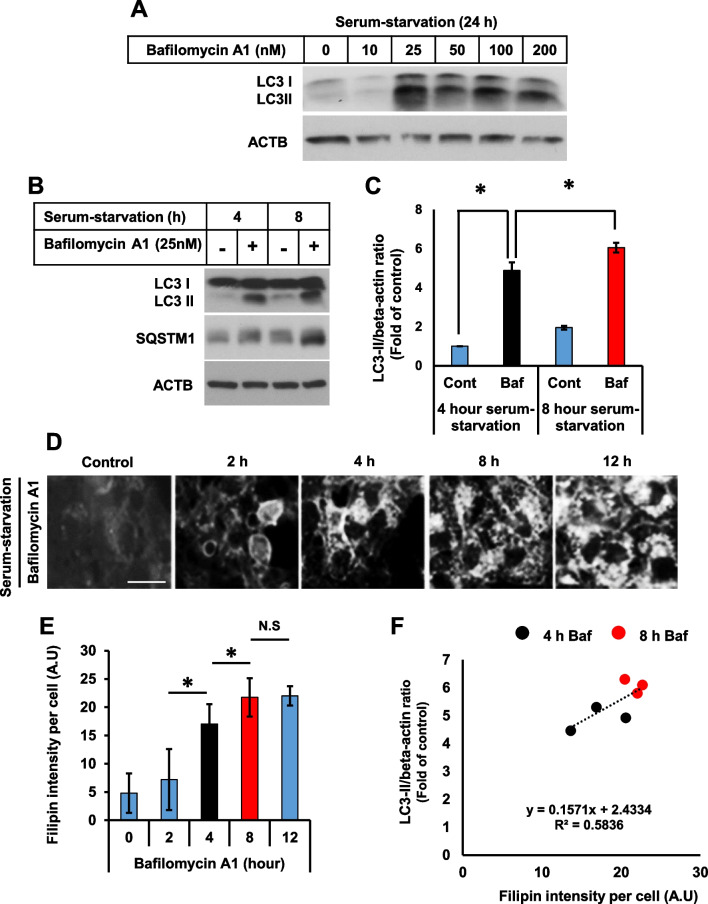
Induction of lysosomal cholesterol sequestration by bafilomycin A1 significantly correlates with autophagy impairment. **A** Cells were treated with different concentrations of bafilomycin A1 in a serum-starvation medium for 24 h. Cells were then subjected to Western blot for LC3 and ACTB. **B** Cells were treated with bafilomycin A1 (25 nM) in a serum-starvation medium for 4 h and 8 h. Cells were then subjected to Western blot for LC3 and ACTB. **C** Intensity of the bands from **B** was quantified using Image J software, and the LC3-II/ACTB ratio was determined. Bar graph represents the mean ± SD (n = 3 experiments). *P < 0.05, Student's t-test. **D** Cells were treated with bafilomycin A1 (25 nM) in a serum-starvation medium for a different duration. Cells were then subjected to filipin staining. Scale bar, 10 μm. **E** Fluorescence intensity of filipin was measured using ImageJ software. Bar graph represents the mean ± SD (n = 3 experiments). Filipin intensity from 50 cells was acquired for each experimental group. Bar graph represents the mean ± SD (n = 3 experiments). *P < 0.05, Student's t-test. **F** Pearson’s correlation coefficient for the association between impaired autophagy flux and cholesterol accumulation between 4 and 8 h in the presence of bafilomycin A1 was determined by plotting individual LC3-II/ACTB ratio and filipin intensity from three different experiments

We also found that 25 nM of bafilomycin A1 efficiently induced LC3-II accumulation. Increasing the concentrations of bafilomycin A1 up to 200 nM did not further alter LC3-II protein expression, indicating that autophagosome accumulation was saturated in cells treated with 25 nM of bafilomycin A1 for 24 h (Fig. 3A). Treatment with bafilomycin A1 efficiently accumulated LC3-II in an early time point at 4 h, which further augmented at 8 h (Fig. 3B, C). In addition, the enhanced intensity of filipin was observable as early as at 4 h, which was saturated at 8 h, suggesting that bafilomycin A1 significantly induced the cholesterol accumulation in a time-dependent manner (Fig. 3D, E). Furthermore, Pearson’s correlation coefficient between an increase in autophagosomes and lysosomal cholesterol accumulation at 4 h and 8 h was significantly correlated, with r^2^ = 0.5836 (Fig. 3F) in bafilomycin A1 treated cells.

### Genetic ablation of autophagy did not result in lysosomal cholesterol accumulation

Although lysosomal cholesterol accumulation in NPC1 deficient cells impairs autophagy flux (10), the impact of genetic ablation of autophagy-related genes, such as ATG5, on lysosomal cholesterol accumulation remains uncertain and controversial (7, 9).

**Fig. 4:**
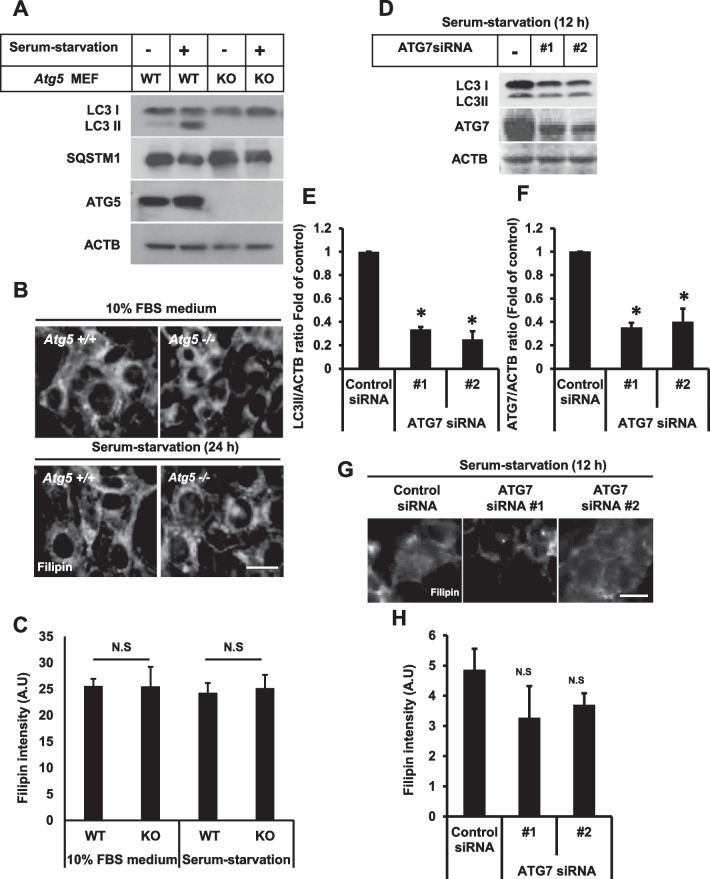
Genetic ablation of autophagy does not induce lysosomal cholesterol accumulation. **A** WT and ATG5 KO MEFs were incubated in either a serum-supplemented medium or serum-starvation medium for 24 h. Cells were then subjected to Western blot for LC3, ATG5, and ACTB. **B** MEFs were incubated in either a serum-supplemented medium or serum-starvation medium for 24 h. Cells were then subjected to filipin staining. Scale bar, 10 μm. **C** Fluorescence intensity of filipin was measured using ImageJ software. Filipin intensity from 50 cells was acquired for each experimental group. Bar graph represents the mean ± SD (n = 3 experiments). *P < 0.05, Student's t-test. **D** RPE1 cells were transfected with control siRNA or ATG7 siRNAs for 48 h followed by further incubation in serum-starvation medium for an additional 12 h. Cells were subjected to Western blot for LC3, ATG7, and ACTB. **E, F** Intensity of the bands from **D** was quantified using Image J software. LC3-II/ACTB ratio **E** and ATG7/ACTB ratio **F** were determined. Bar graph represents the mean ± SD (n = 3 experiments). *P < 0.05, Student's t-test. **G** RPE1 cells were transfected with control siRNA or ATG7 siRNAs for 48 h followed by further incubation in serum-starvation medium for an additional 12 h. Cells were subjected to filipin staining. Scale bar, 10 μm. **H** Fluorescence intensity of filipin was measured using the ImageJ software. Filipin intensity from 50 cells was acquired for each experimental group. Bar graph represents the mean ± SD (n = 3 experiments). *P < 0.05, Student's t-test

Mouse embryonic fibroblasts (MEFs) from ATG5 wild-type (WT) and ATG5 knockout (KO) mice were stained with filipin in either serum-fed or serum-starved conditions (Fig. 4A–C) to determine whether autophagy impairment results in lysosomal cholesterol accumulation. We observed that the basal level of filipin intensity was very high in ATG5 WT and KO MEFs in both serum-fed and serum-starved conditions, suggesting that autophagy impairment caused by defective autophagosome formation might not result in lysosomal cholesterol accumulation. Furthermore, MEFs are enriched with free cholesterol in the cytosolic perinuclear compartment and the plasma membrane, where filipin staining observed as a punctate structure and cell boundary was not really cholesterol (23). This finding contradicts Sarkar et al. that highlight WT MEFs do not show extensive filipin staining, while autophagy deficiency enhances lysosomal cholesterol accumulation (9). Furthermore, studies have reported that filipin-stained punctate structure decreased in ATG5 KO MEFs, even in the presence of U18666A (11), directly contradicting Sarkar et al. (9). Therefore, a high level of free cholesterol stained by filipin does not signify the impaired transport of intracellular cholesterol in either ATG5 WT or KO MEFs. Furthermore, ATG7 depletion significantly decreased LC3-II protein expression (Fig. 4D–F), suggesting that autophagosome formation of RPE1 cells was decreased in serum-starved condition without enhancing filipin intensity (Fig. 4G, H). These observations suggest that genetic ablation of autophagy does not necessarily result in lysosomal cholesterol accumulation.

### U18666A and bafilomycin A1 are potent inhibitors of intracellular cholesterol transport

**Fig. 5:**
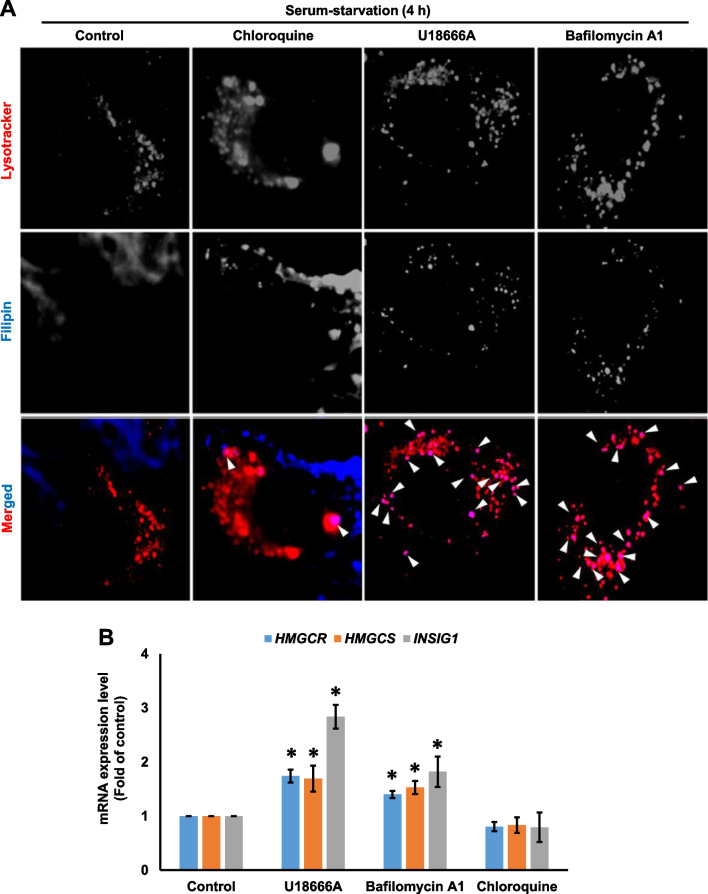
U18666A and BafilomycinA1 are potent inhibitors of intracellular cholesterol transport. **A** RPE1 cells were treated with U18666A (2 μg), bafilomycin A1 (25 nM), or chloroquine (5 μM) in a serum-starvation medium for 4 h. Cells were stained with filipin: scale bar, 10 μm. Arrowheads represent filipin-laden lysosomes **B** RPE1 cells were treated with U18666A (2 μg), bafilomycin A1 (25 nM), or chloroquine (5 μM) in a serum-starvation medium for 24 h. **B** Total RNA was extracted, and the expression of *HMGCR*, *HMGCS*, and *INSIG1* was analyzed using a quantitative real-time polymerase chain reaction (qPCR). The bar graph represents the mean ± SD (n = 3 experiments). *P < 0.05, Student's t-test

As an increase in the early accumulation of autophagosomes and filipin intensities was significantly correlated in the presence of U18666A and bafilomycin A, we tested whether cholesterol was early accumulated in lysosomal compartment. The results showed that filipin-positive lysotracker puncta increased after 4 h of U18666A or bafilomycin A1 treatment, suggesting that cholesterol was exclusively accumulated in the lysosomes (Fig. 5A). Contrastingly, filipin-positive lysotracker puncta was not observed in the presence of chloroquine at 4 h (Fig. 5A).

In addition, SREBP2 target genes, including *HMGCR*, *HMGCS*, and *INSIG1*, were significantly upregulated by U18666A and bafilomycin A1, indicating the high potency of inhibition of intracellular cholesterol transport might work (Fig. 5B). Chloroquine did not upregulate SREBP2 target genes, suggesting that it was not an inhibitor of intracellular cholesterol transport. Thus, impaired autophagy flux might not necessarily result in lysosomal cholesterol accumulation. Contrastingly, the rate of lysosomal cholesterol accumulation and autophagosome accumulation in the presence of potent inhibitors for intracellular cholesterol transport was significantly correlated.

### U18666A and bafilomycin A1 prevent syntaxin17 trafficking to autophagosome

**Fig. 6:**
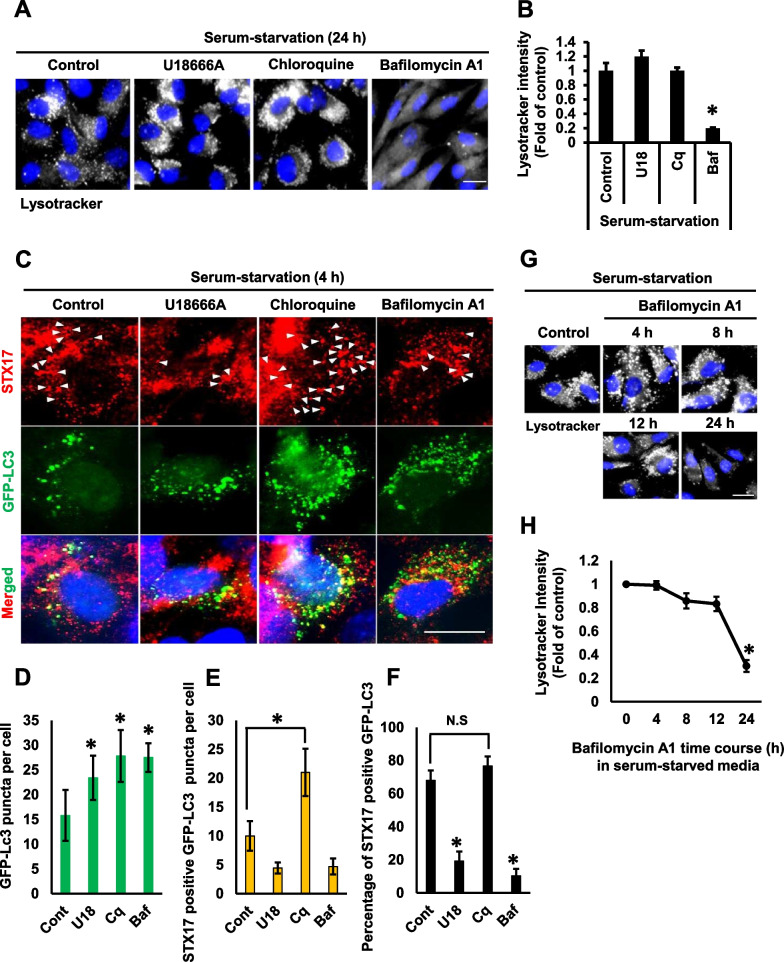
U18666A and bafilomycin A1 impairs Syntaxin17 trafficking to autophagosomes. **A** RPE1 cells were treated with U18666A (2 μg), chloroquine (5 μM), or bafilomycin A1 (25 nM) for 24 h in a serum-free medium. Cells were then subjected to lysotracker staining. Scale bar, 10 μm. **B** Intensities from 50 cells were acquired for each experimental group using ImageJ software. The bar graph represents the mean ± SD (n = 3 experiments). *P < 0.05, Student's t-test. **C** Cells were transfected with GFP-LC3 for 12 h in serum-supplemented media. Cells were then treated with U18666A (2 μg), chloroquine (5 μM), or bafilomycin A1 (25 nM) for 4 h in a serum-starvation medium followed by 4% paraformaldehyde fixation and immunostaining with STX17 antibody. Scale bar, 10 μm. Arrowheads represent STX17 trafficking to autophagic structures (GFP-LC3). **D** Quantification of GFP-LC3 from 50 transfected cells was analyzed for each experimental group. The bar graph represents the mean ± SD (n = 3 experiments). *P < 0.05, Student's t-test. **E** Determination of the number of STX17 positive GFP-LC3 puncta. Fifty transfected cells were analyzed for each experimental group. The bar graph represents the mean ± SD (n = 3 experiments). *P < 0.05, Student's t-test. **F** Percentage of STX17 positive GFP-LC3 puncta were calculated from **D** and **E**. The bar graph represents the mean ± SD (n = 3 experiments). *P < 0.05, Student's t-test. **G** Cells were treated with bafilomycin A1 (25 nM) for different time points in serum-starvation medium. Cells were then subjected to Lysotracker staining. Scale bar, 10 μm. **H** Lysotracker intensities from 50 cells were acquired using the ImageJ software for each experimental group. The bar graph represents the mean ± SD (n = 3 experiments). *P < 0.05, Student's t-test

As lysosomal cholesterol accumulation by chloroquine and bafilomycin A1 has been linked to the prevention of lysosomal acidification (17), we speculated that an association among lysosomal pH, autophagy impairment, and lysosomal cholesterol accumulation might associate together. Therefore, lysosomal acidity was analyzed using pH-sensitive lysosomal dye, LysoTracker Red, in the presence of chloroquine, U18666A, and bafilomycin A1. As expected, treatment with bafilomycin A1 (25 nM) for 24 h dramatically decreased lysosomal acidity evidenced by a significant decrease in LysoTracker Red puncta (Fig. 6A, B). However, chloroquine (5 μM) did not alter LysoTracker Red puncta, indicating lysosomal pH was not changed (Fig. 6A, B). This result agrees with a recent study highlighting that chloroquine does not prevent lysosomal acidification up to 200 μM in U2OS cells (16). Although treatment with U18666A resulted in massive cholesterol accumulation (Fig. 2A), it did not prevent lysosomal acidification (Fig. 6A, B), suggesting that the prevention of lysosomal acidification is independent of lysosomal cholesterol accumulation. Although further investigation is required to elucidate the inhibitory mechanism of intracellular cholesterol transport by bafilomycin A1, lysosomal acidity and lysosomal cholesterol accumulation may be independent processes related with the different properties of the same compound.

Impaired autophagy flux in NPC1 disease is resulted from a failure of SNARE proteins to perform autophagosome–lysosome fusion (9, 10, 24). As both bafilomycin A1 and U18666A caused lysosomal cholesterol accumulation, irrespective of the status of lysosomal pH, we speculated whether the inhibition of autophagy flux is associated with defective autophagosome–lysosome fusion. STX17 is a SNARE protein that drives autophagosome–lysosome fusion. Its presence on an autophagosome strongly suggests that an autophagosome is matured and competent to fuse with lysosome (25). Therefore, we analyzed whether autophagosomes accumulated in the presence of three compounds are competent for fusion by comparing the percentage of STX17 positive autophagosomes. As shown in Fig. 3C and F, serum starvation triggered the formation of autophagosomes within 4 h, at which point the most of autophagosomes was positive for STX17. Both U18666A and bafilomycin A1 severely reduced the presence of STX17 positive autophagosomes, suggesting the defective trafficking of STX17 (Fig. 6C–F). Notably, an accumulation of STX17 positive autophagosomes was increased in cells treated with chloroquine. This is in agreement with a previous report highlighting that chloroquine does not affect STX17 trafficking to autophagosomes (16). Many numbers of GFP-LC3 puncta per cell were shown in cells treated with drugs compared to the control cells, indicating that STX17 trafficking is an early event associated with autophagic flux. In addition, the defective STX17 trafficking might be responsible for impaired autophagosome–lysosome fusion, thereby resulting in autophagosome accumulation in the presence of both U18666A and bafilomycin A1.

A time-course study was performed to analyze lysosomal acidity and verify whether the defective STX17 trafficking, observed in the presence of bafilomycin A1, did not prevent lysosomal acidification. The data showed that bafilomycin A1 did not prevent lysosomal acidification for up to 12 h (Fig. 6G, H). Therefore, the early accumulation of autophagosomes, induced by bafilomycin A1, was found to be due to impaired autophagosome–lysosome fusion, possibly caused by a failure of STX17 trafficking to autophagosomes. Thus, these data support that STX17 trafficking is impaired in the presence of U18666A and bafilomycin A1, but not chloroquine.

### Methyl-β-cyclodextrin efficiently rescues STX17 trafficking to autophagosome–lysosome fusion by U18666A and Bafilomycin A1

It has been reported that methyl-β-cyclodextrin (MβCD) recovers the impaired autophagy flux in NPC1 disease by activating interactions among STX17, VAMP8, and SNAP29 for autophagosome–lysosome fusion (10). We speculated whether MβCD could rescue the impaired autophagic flux, observed in cells treated with U18666A and bafilomycin A1 through the clearance of accumulated cholesterol from lysosomal compartments.

We also confirmed that treatment with 10 μM MβCD for 24 h efficiently enhanced LC3-II levels in the serum-starved condition without altering SQSTM1/p62 levels (Additional file 1: Fig. S1A). Furthermore, a lower concentration of MβCD did not affect mTORC1 activation state, suggesting that MβCD activates autophagosome formation without activating mTORC1 pathway. In addition, 20 μM of MβCD was sufficient to clear cholesterol accumulated in the presence of either U18666A or bafilomycin A1 (Additional file 1: Fig. S1B–D).

Colocalization of lysosome marker LAMP1 to GFP-LC3 was tested to confirm whether MβCD could rescue the impaired autophagic flux by enhancing autophagosome–lysosome fusion in the presence of U18666A or bafilomycin A1. Interestingly, GFP-LC3 was found to be extensively colocalized with LAMP1 in the control cells (Additional file 1: Fig S2A, B), indicating that GFP signal quenching inside lysosomes might require longer duration. Both U18666A and bafilomycin A1 significantly decreased the colocalization of LAMP1 to GFP-LC3 (Additional file 1: Fig S2A, B), implicating that autophagosome–lysosome fusion was potentially inhibited because of the impaired STX17 trafficking to autophagosomes (Fig. 6C–F). Although chloroquine did not affect STX17 trafficking, it efficiently prevented autophagosome–lysosome fusion (Additional file 1: Fig S2 A, B) as suggested by Mauthe et al., shown that the prevention of autophagosome–lysosome fusion by chloroquine is independent of STX17 trafficking to autophagosomes (16). While MβCD significantly increased the colocalization of LAMP1 to GFP-LC3 in the presence of U18666A or bafilomycin A1 (Additional file 1: Fig S2A, B), MβCD did not improve the impaired autophagosome–lysosome fusion in the presence of chloroquine (Additional file 1: Fig S2A, B), indicating that the impaired fusion of autophagosome–lysosome by chloroquine is not directly related to lysosomal cholesterol accumulation.

**Fig. 7:**
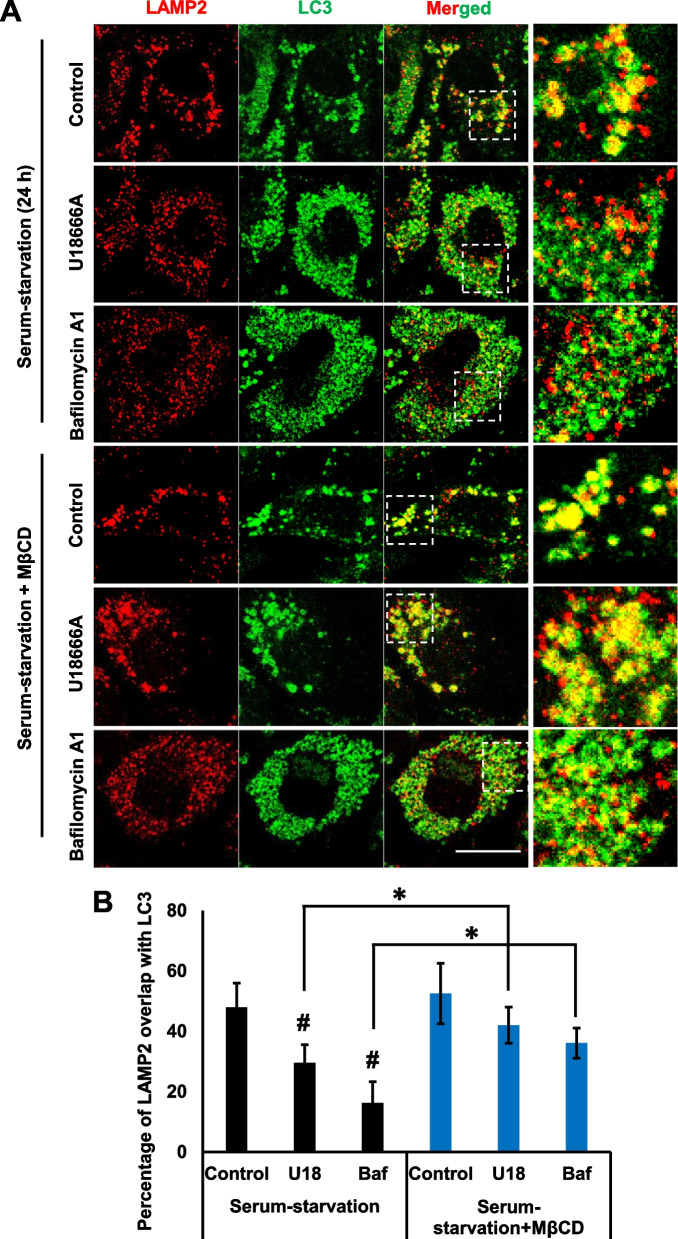
Methyl- β-cyclodextrin efficiently rescues impaired autophagosome-lysosome fusion by U1866A and bafilomycin A1. **A** RPE1 cells were treated with U18666A (2 μg) or bafilomycin A1 (25 nM) for 12 h in a serum-starved medium. Cells were further incubated in the presence or absence of MβCD (20 μM) for additional 24 h without changing the medium. Cells were then fixed with 4% paraformaldehyde and immunostained for LAMP2 and LC3. Scale bar, 10 μm. **B** The quantification of GFP-LC3 puncta overlapping with LAMP1 from 30 transfected cells using ImageJ software was determined. Bar graph represents the mean ± SD (n = 3 experiments). #*P* < 0.05, Student's t-test (test groups were compared with control). *P < 0.05 Student's t-test (respective test groups were compared in the presence or absence of MβCD)

To further confirm whether lysosomal cholesterol accumulation impairs autophagosome–lysosome fusion and MβCD ameliorates autophagosome–lysosome fusion, the colocalization of endogenous LC3 puncta with lysosome marker LAMP2 was analyzed. Level of LAMP2 colocalization with LC3 was decreased in U18666A and bafilomycin A1 treated cells compared to the control serum-starved cells (Fig. 7A, B). Although the population of LAMP2 puncta was small, its size was considerably larger when it colocalized with LC3 puncta, as in the control serum-starved cells (Fig. 7A, B). Contrastingly, the population of LAMP2 and LC3 puncta is more abundant and smaller in the presence of U18666A and bafilomycin A1 compared to the control serum-starved cells (Fig. 7A, B), implicating impaired autophagic flux happened. Thus, the data suggest that many of lysosomes might fuse with autophagosome in the control serum-starved cells, while the fusion of lysosomes to autophagosomes is impaired in cells in the presence of U18666A and bafilomycin A1.

MβCD stimulated the formation of larger LAMP2 puncta colocalized with endogenous LC3 puncta in cells treated with U18666A (Fig. 7A, B), suggesting the restoration of impaired autophagosome–lysosome fusion. Moreover, the population of LC3 puncta was drastically reduced by MβCD in the presence of U18666A, suggesting the impaired autophagy flux was ameliorated (Fig. 7A, B). Although the effect of MβCD on the formation of larger LAMP2 puncta by bafilomycin A1 was not drastic, MβCD stimulated the localization of the majority of LAMP2 population to LC3 puncta. MβCD did not decrease LC3 puncta in the presence of bafilomycin A1, implying that the impaired autophagy flux was not rescued. Taken together, MβCD efficiently ameliorates the impaired process of autophagosome–lysosome fusion.

### Minimum concentration of bafilomycin A1 does not induce ER stress

**Fig. 8:**
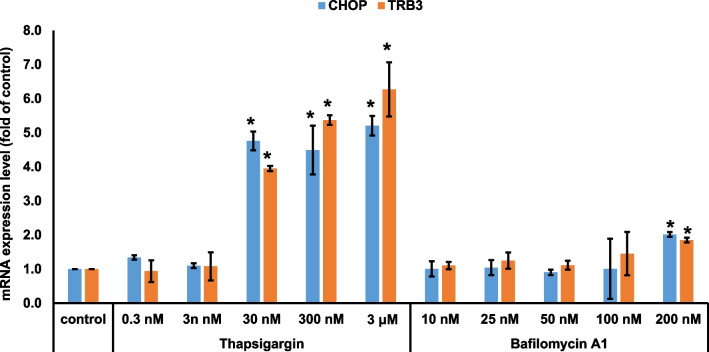
Minimum concentration of bafilomycin A1 does not induce ER stress. RPE1 cells were treated with different concentrations of either thapsigargin or bafilomycin A1 in a serum-starvation medium for 24 h. RNAs were extracted, and the expression of *CHOP* and *TRB3* was analyzed using quantitative real-time polymerase chain reaction (qPCR). Bar graph represents the mean ± SD (n = 3 experiments). *P < 0.05, Student's t-test

A recent study showed that SERCA inhibition and cytosolic Ca^2+^ accumulation using a high concentration of bafilomycin A1 (200 nM) is directly related to the impaired autophagosome–lysosome fusion (19). An important feature of SERCA inhibition and cytosolic Ca^2+^ accumulation is known as the induction of ER stress and the inhibition of autophagy flux resulting from the blockage of autophagosome fusion with endocytic system (26). mRNA expression of ER stress markers, CHOP and TRB3, was tested to determine the range of concentrations for ER stress caused by bafilomycin A1. As shown in Fig. 8, 30 nM of thapsigargin was sufficient to induce robust ER stress. mRNA expression levels of CHOP and TRB3 were not altered up to 100 nM bafilomycin A1, however they were slightly increased at 200 nM bafilomycin A1. Although high concentrations of bafilomycin A1 could influence ER stress, we suggest that bafilomycin A1 directly prevents autophagosome–lysosome fusion by impairing of STX17 trafficking to autophagosomes because of lysosomal cholesterol accumulation and no relevance to ER stress.

### Methyl- β-cyclodextrin rescues impaired autophagy flux by U18666A

**Fig. 9:**
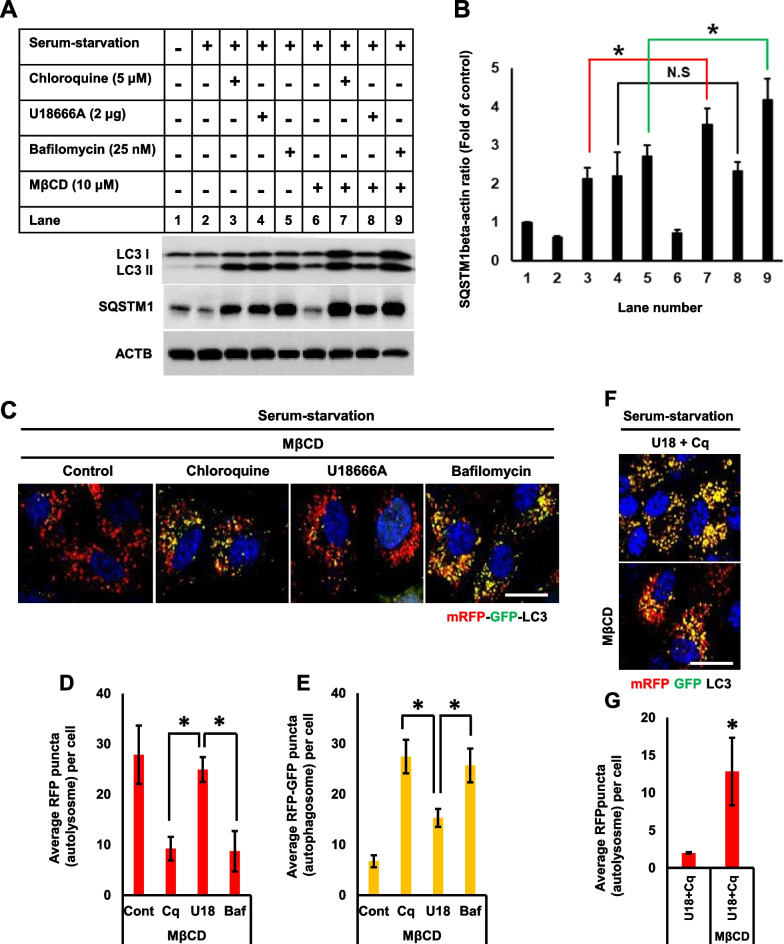
Methyl- β-cyclodextrin efficiently rescues impaired autophagy flux in the presence of U18666A. **A** Cells were treated with U18666A (2 μg), chloroquine (5 μM), or bafilomycin A1 (25 nM) for 12 h in a serum-starvation medium. Cells were further incubated in the presence or absence of MβCD (20 μM) for an additional 24 h without changing medium and subjected to Western blot for LC3, SQSTM1, and ACTB. **B** Intensity of the bands from **A** was quantified using the ImageJ software, and the SQSTM1/ACTB ratio was determined. Bar graph represents the mean ± SD (n = 3 experiments). *P < 0.05, Student's t-test. **C** RPE1-mRFP-GFP-LC3 cells were treated as in **A**, fixed with 4% paraformaldehyde, and stained with DAPI. Scale bar, 10 μm. **D, E**, Autophagy status was determined by counting autolysosomes (punctate structures with only RFP signal) or autophagosomes (yellow puncta with both GFP and RFP signal). At least 100 cells were counted for each experimental group. Bar graph represents the mean ± SD (n = 3 experiments). *P < 0.05, Student's t-test. **F** RPE1-mRFP-GFP-LC3 cells were co-treated with U18666A (2 μg) and chloroquine (5 μM) for 12 h in a serum-starvation medium. Cells were further incubated in the presence or absence of MβCD (20 μM) for an additional 24 h without changing medium, fixed with 4% paraformaldehyde, and stained with DAPI. Scale bar, 10 μm. **G** Autophagy status was determined by counting autolysosomes (punctate structures with only RFP signal). At least 100 cells were counted for each experimental group. The bar graph represents the mean ± SD (n = 3 experiments). *P < 0.05, Student's t-test

We further analyzed SQSTM1 (p62) accumulation to test whether MβCD rescues autophagy flux in the presence of three drugs. MβCD increased the level of LC3-II protein without altering p62 levels in the serum-starved control cells (Fig. 9A, B), implicating that MβCD increased autophagosome formation without altering autophagy flux. However, the level of LC3-II protein was decreased by MβCD in the presence of U18666A (but still higher compared to the control cells treated with MβCD) without altering p62 levels, indicating that autophagy flux was increased, potentially due to the activation of autophagosome–lysosome fusion. MβCD further increased the protein levels of both LC3-II and p62 in the presence of chloroquine and bafilomycin A1, suggesting that MβCD increased autophagosome formation, however autophagy flux was still inhibited. These results indicate that the inhibition of autophagy flux by chloroquine is independent of lysosomal cholesterol accumulation. Although MβCD rescued impaired autophagosome–lysosome fusion in the presence of bafilomycin A1, autophagy flux was not improved because of the ability of bafilomycin A1 to prevent lysosomal acidification over longer periods of treatment (Fig. 6G, H).

Autophagy flux was analyzed in mRFP-GFP-LC3 RPE1 stable cells to confirm whether MβCD could rescue the impaired autophagy flux by U18666A but not by both chloroquine and bafilomycin A1. MβCD increased autophagy flux in the presence of U18666A, as suggested by comparable numbers of autolysosomes (RFP-LC3) seen in the control cells (Fig. 9C–E). However, autophagosomes (GFP-RFP-LC3) were not cleared by MβCD in the presence of both chloroquine and bafilomycin A1, indicating the continued impairment of autophagy flux (Fig. 9C–E). As mentioned, both chloroquine and U18666A impaired autophagy through different mechanisms. Thus, co-treatment with these drugs severely reduced autolysosomes (Fig. 1E, G). However, MβCD significantly increased autolysosomal population in cells co-treated with chloroquine and U18666A (Fig. 9F, G), suggesting that MβCD successfully recovered a part of the impaired autophagy flux caused by U18666A.

## Discussion

This study highlights the mechanism by which inhibitors of intracellular cholesterol transport undermine autophagosome–lysosome fusion by preventing STX17 trafficking to autophagosomes, irrespective of lysosomal acidity. Cholesterol accumulation in the lysosome interferes with cholesterol-dependent functions of endosomes and impairs vesicle trafficking (27–30). Thus, the impaired autophagy flux, observed in the presence of U18666A or bafilomycin A1, may attribute to defective autophagosome–lysosome fusion, directly resulting from cholesterol accumulation in the lysosome. Chloroquine was found to prevent autophagy flux by inhibiting autophagosome–lysosome fusion neither preventing STX17 trafficking to autophagosomes nor preventing lysosomal acidification. This finding agrees with a previous report stating that chloroquine prevents autophagosome–lysosome fusion through the defective trafficking of SNAP29 to autophagosomes without impairment of STX17 trafficking (16). Here, inhibitors of intracellular cholesterol transport, including U1866A and bafilomycin A1, impair autophagosome–lysosome fusion in a manner different from chloroquine, which does not affect intracellular cholesterol transport (Fig. 1C–F). Moreover, MβCD activates autophagosome–lysosome fusion through the mobilization of cholesterol accumulated in the presence of U18666A or bafilomycin A1, but not in the presence of chloroquine. This discrepancy suggests that intracellular cholesterol transport regulates specific steps in the process of autophagosome–lysosome fusion. A recent study reported that lysosomal cholesterol accumulation prevents ciliogenesis through a selective impairment of Rab8 trafficking to the centrioles (31). Although both bafilomycin A1 and U18666A prevent the primary ciliogenesis by defective trafficking of Rab8, the effect of impaired autophagic flux on ciliogenesis cannot be excluded (32, 33). Although further investigation is required, we speculate that U18666A and bafilomycin A1 might selectively affect cholesterol-dependent trafficking of vesicles, such as STX17-positive and Rab8-positive vesicles, affecting the related cellular processes they regulate. In addition, certain proteins involved in vesicular fusion events might be sensitive to cholesterol distribution within cells.

Chloroquine has been reported to increase lysosomal pH even at 1 μM in peritoneal macrophages (34). However, chloroquine was not found to effectively decrease LysoTracker Red fluorescence in this study, suggesting that it might not alter lysosomal acidification. Whereas several studies using the pH-sensitive dye demonstrate that chloroquine drastically decreases LysoTracker Red-positive puncta (35–38). However, others show that chloroquine does not alter lysosomal acidity (16, 39–41). Although LysoTracker Red can only be used to estimate lysosomal acidity, the discrepancies observed in chloroquine's ability to alter lysosomal acidification might be associated with different cell types rather than chloroquine concentrations used. Although the possibility of a very high concentration of chloroquine eliciting a nonspecific effect on both lysosomal cholesterol accumulation and lysosomal acidity cannot be excluded (13), we used the minimum concentration of chloroquine in this study that does not alter lysosomal acidity or result in lysosomal cholesterol accumulation. In addition, the minimum concentration of bafilomycin A1 used in this study did not affect lysosomal acidity even though lysosomal cholesterol accumulation occurred. Taken together, we propose that lysosomal acidification and cholesterol accumulation are independent in the processes of autophagy.

Bafilomycin A1 significantly reduces STX17 positive autophagosomes owing to STX17 disengagement from an autophagosome after fusion with the lysosomes, implying that the STX17 mediated fusion of autophagosomes with the lysosomes is not affected by bafilomycin A1 (16). This interpretation contradicts our observation that bafilomycin A1 prevents STX17 trafficking to autophagosomes. This study highlights that despite low autophagosomal counts in the serum-starved control cells, over 60% of cells were positive for STX1, implying that, at any given time point, over half of existing autophagosomes are positive for STX17, even though the disengagement of STX17 from autophagosomes after fusion with the lysosomes has been described as a dynamic and rapid process (27). Therefore, there could be a similar percentage of STX17 positive autophagosomes per cell in the presence of bafilomycin A1 and in the control starved cells at any given time points when STX17 is transferred to autophagosomes. Thus, a decrease in the percentage of STX17 positive autophagosomes in the presence of bafilomycin A1 is possibly due to trafficking defects rather than the rapid disengagement of STX17 from autophagosomes after fusion with lysosomes.

**Fig. 10:**
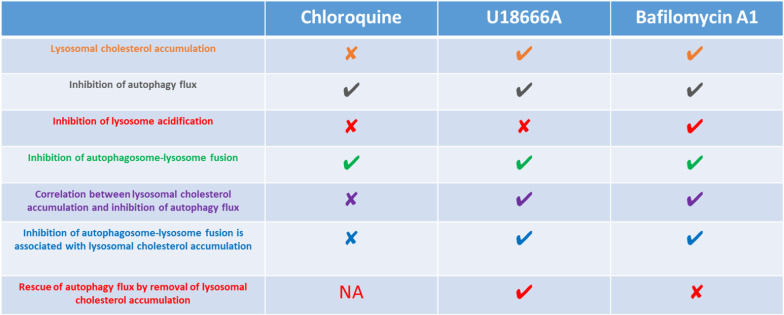
Descrimination of Chloroquine, U1866A and Bafilomycin A on autophagy and cholesterol accumulation

The notion that bafilomycin A1 decreases the colocalization of the lysosomes with autophagosomes contradicts to a previous report (16). This could be because of the exclusion of positive control for autophagy flux, as serum-starvation presented in the previous report, indicating that a number of autophagosomes colocalized with the lysosomes was similar to the control cells under basal conditions (16). The inclusion of the positive control for autophagy flux is essential to compare the instances when pharmacological inhibitors of autophagy tend to increase the lysosome numbers and the accumulation of autophagosomes, as shown in a previous report (16). The clear discrimination of three pharmacological inhibitors is described in Fig. 10.

## Conclusion

Our results indicate that the impaired autophagy flux does not necessarily result in lysosomal cholesterol accumulation. However, lysosomal cholesterol accumulation invariably inhibits autophagy flux caused by defective lysosome-autophagosome fusion, potentially due to the impaired STX17 trafficking to autophagosomes. Our results would be beneficial in studies focusing to understand the mechanism of autophagy in detail among the relationships involving, autosome, autophagosome, and autolysosome. However, lysosomal cholesterol accumulation by compounds which inhibit autophagosome–lysosome fusion needs to be analyzed further. Taken together, each of autophagy inhibitors including chloroquine, bafilomycin A1, and U18666A, is different in the induction mechanism of autophagy which is also orchestrated by autophagosome and lysosome (Fig. 10).

## Supplementary Information

**Additional file 1: Fig. S1**. Methyl-β-cyclodextrin efficiently clears accumulated cholesterol in the presence of U18666A and bafilomycin A1. **A** RPE1 cells were treated with or without different concentration of MβCD in a serum-starvation medium for 24 hours. Cells were then subjected to Western blot for LC3, SQSTM1, phospho-RPS6, RPS6, and ACTB. **B** Cells were either treated with U18666A (2 μg) or bafilomycin (25 nM) for 12 hours in a serum-starvation medium. Cell were further incubated for additional 24 hours or incubated for additional 24 hours with MβCD (10 μM or 20 μM) without changing the medium. Cells were then subjected to filipin staining. Scale bar, 20 μm. **C**, **D** Fluorescence intensities of filipin were measured by ImageJ Software. Filipin intensity from 50 cells were acquired for each experimental group. Bar graph represents mean ± SD (n = 3 experiments). *P < 0.05, Student's t-test. **Fig. S2**. Methyl-β-cyclodextrin enhances LAMP1 localization to GFP-LC3 positive structure in the presence of U18666A and bafilomycin A1. **A** RPE1 cells were treated with U18666A (2 μg), chloroquine (5 μM) or bafilomycin A1 (25 nM) for 12 hours in a serum-starvation medium. Cells further incubated with or without MβCD (20 μM) for additional 24 hours without changing medium. GFP-LC3 transfection transfection was carried out and after 6 hours of transfection cells were fixed by 4% paraformaldehyde and immunostained with LAMP1 antibody. Scale bar, 10 μm. **B** Quantification of GFP-LC3 puncta overlapping with LAPM1 was quantified from 30 transfected cells by using ImageJ software. Bar graph represents mean ± SD (n = 3 experiments). #*P* < 0.05, Student's t-test (test group were compared with control). *P < 0.05 Student's t-test (respective test groups were compared in the presence or absence of MβCD).

## Data Availability

All data generated and/or analyzed in this study are available from the corresponding author on reasonable request.

## Abbreviations

SNARE: Soluble N-ethylmaleimide-sensitive factor attachment protein receptor
STX17: Syntaxin 17
SNAP29: Synaptosomal-associated protein 29
NPC: Niemann Pick type C
SERCA: Sarco/endoplasmic reticulum Ca^2+^-ATPase
PCC: Pearson’s correlation coefficient
MβCD: Methyl-β-cyclodextrin
