# Supplementary material for: Intracellular cholesterol transport inhibition Impairs autophagy flux by decreasing autophagosome–lysosome fusion
Source: Cell Commun Signal. 2022 Nov 25;20:189. doi: 10.1186/s12964-022-00942-z (PMC9701069; doi:10.1186/s12964-022-00942-z)
Supplement: Supplementary file 2 — Additional file 1: Fig. S1. Methyl-β-cyclodextrin efficiently clears accumulated cholesterol in the presence of U18666A and bafilomycin A1. A RPE1 cells were treated with or without different concentration of MβCD in a serum-starvation medium for 24 hours. Cells were then subjected to Western blot for LC3, SQSTM1, phospho-RPS6, RPS6, and ACTB. B Cells were either treated with U18666A (2 μg) or bafilomycin (25 nM) for 12 hours in a serum-starvation medium. Cell were further incubated for additional 24 hours or incubated for additional 24 hours with MβCD (10 μM or 20 μM) without changing the medium. Cells were then subjected to filipin staining. Scale bar, 20 μm. C, D Fluorescence intensities of filipin were measured by ImageJ Software. Filipin intensity from 50 cells were acquired for each experimental group. Bar graph represents mean ± SD (n = 3 experiments). *P < 0.05, Student's t-test. Fig. S2. Methyl-β-cyclodextrin enhances LAMP1 localization to GFP-LC3 positive structure in the presence of U18666A and bafilomycin A1. A RPE1 cells were treated with U18666A (2 μg), chloroquine (5 μM) or bafilomycin A1 (25 nM) for 12 hours in a serum-starvation medium. Cells further incubated with or without MβCD (20 μM) for additional 24 hours without changing medium. GFP-LC3 transfection transfection was carried out and after 6 hours of transfection cells were fixed by 4% paraformaldehyde and immunostained with LAMP1 antibody. Scale bar, 10 μm. B Quantification of GFP-LC3 puncta overlapping with LAPM1 was quantified from 30 transfected cells by using ImageJ software. Bar graph represents mean ± SD (n = 3 experiments). #P < 0.05, Student's t-test (test group were compared with control). *P < 0.05 Student's t-test (respective test groups were compared in the presence or absence of MβCD). [file 12964_2022_942_MOESM2_ESM.docx]

arch 26, 2020

To Whom It May Concern

It gives me an immeasurable delight to recommend one of my students, Mr. Laxman Manandhar

to pursue his further study. I have had the opportunity to know him since 2015 when her joined

this Department for his BPharm study. He graduated in 2019 with a CGPA of 3.48 in the scale of

4.

While undertaking his undergraduate study, Laxman maintained very good academic standing

throughout the period. Laxman had the ability to grasp subject matter quickly. He did well in the

course I have taught. Laxman was an exuberant student with optimism towards development. He

had an avid zeal to develop on his own and he made wholehearted commitment in every aspect of

his work. Also he had very good written and spoken English and he was a student with good

presentation and communication skills.

Laxman was a dynamic student of the University and he possesses both the intellectual mind and

the self-motivated soul that are the essential prerequisites for anyone to develop. I have no doubt

that Laxman has planned carefully and I believe he will definitely accomplish his endeavour. An

opportunity to pursue further studies will enable Laxman to further expand his knowledge in the

field of development and hone his skills.

For further information I can be reached at 977 11 415100.

Yours Truly,

Rajani Shakya, MPharm, PhD

Associate Professor &

Head of the Department

**Supplementary Data to:**

**Yunash Maharjan, Raghbendra Kumar Dutta, Jinbae Son, Batchingis Chinbold, Xiaofan Wei, Hyunsoo Kim, Donghyun Kim, Channy Park and Raekil Park**

**Pharmacological Inhibition of Intracellular Cholesterol Transport Impairs Autophagy Flux by Decreasing Autophagosome-Lysosome Fusion**

**
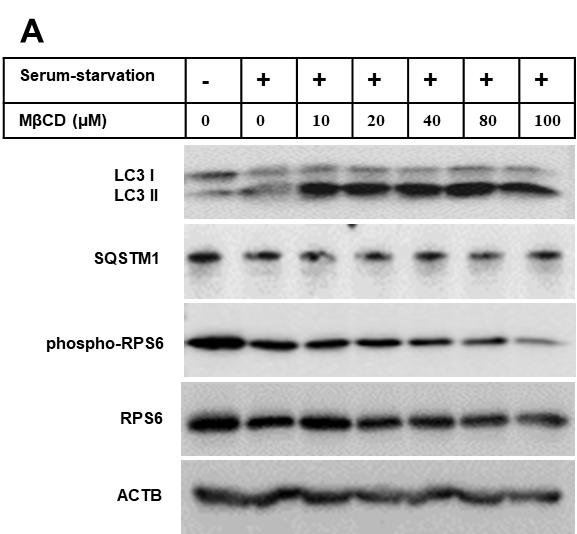

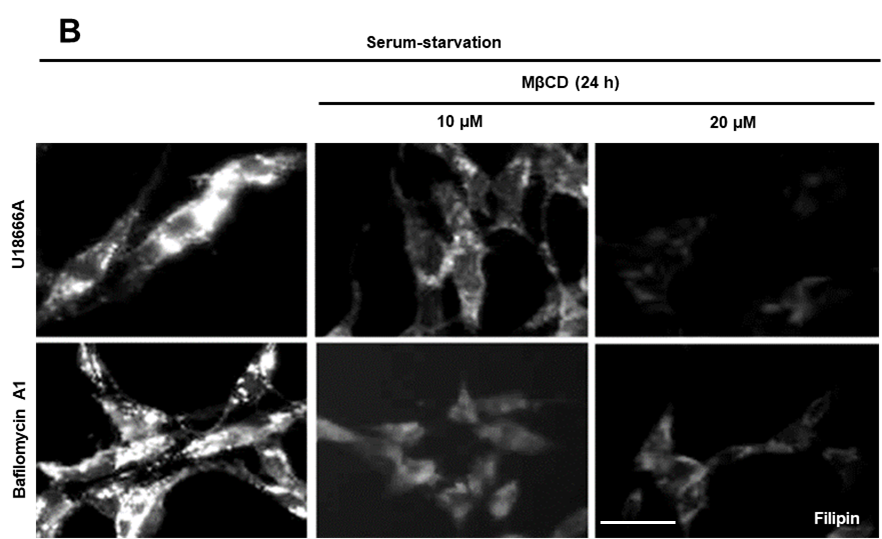
**

**
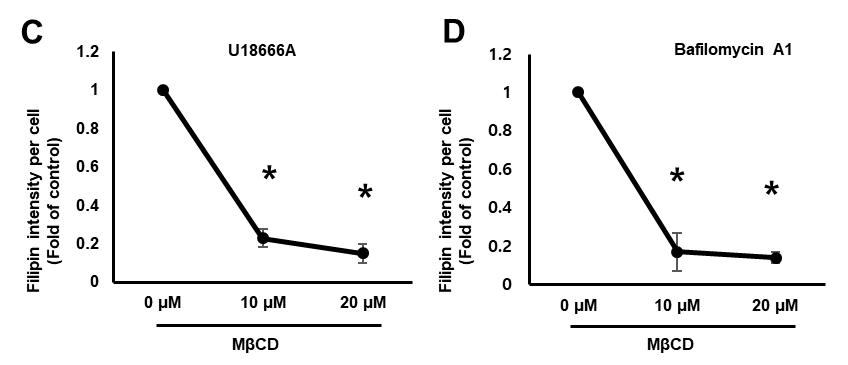
**

**Figure S1. Methyl-β-cyclodextrin efficiently clears accumulated cholesterol in the presence of U18666A and bafilomycin A1.**

**(A)** RPE1 cells were treated with or without different concentration of MβCD in a serum-starvation medium for 24 hours. Cells were then subjected to Western blot for LC3, SQSTM1, phospho-RPS6, RPS6, and ACTB. **(B)** Cells were either treated with U18666A (2 μg) or bafilomycin (25 nM) for 12 hours in a serum-starvation medium. Cell were further incubated for additional 24 hours or incubated for additional 24 hours with MβCD (10 μM or 20 μM) without changing the medium. Cells were then subjected to filipin staining. Scale bar, 20 μm. **(C, D)** Fluorescence intensities of filipin were measured by ImageJ Software. Filipin intensity from 50 cells were acquired for each experimental group. Bar graph represents mean ± SD (n=3 experiments). *P < 0.05, Student's t-test.

**
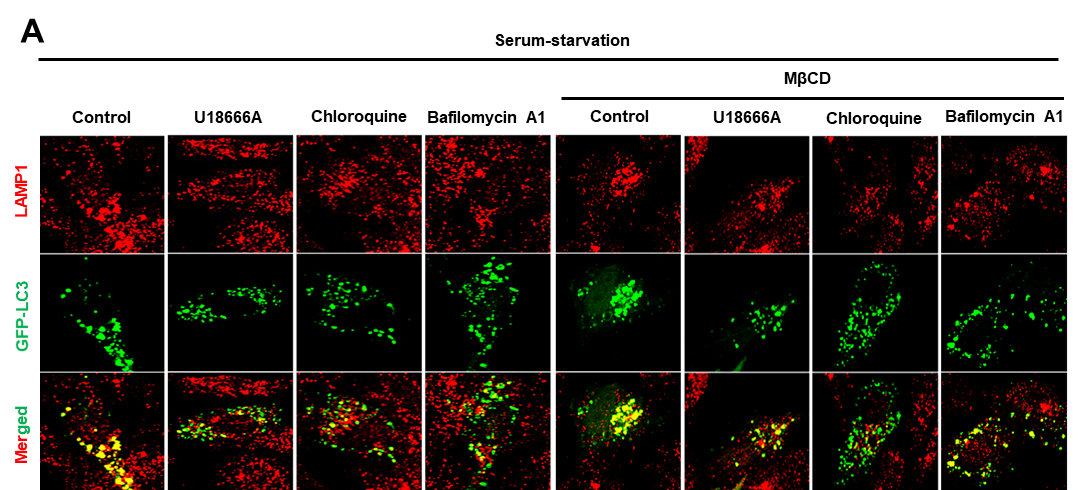
**

**
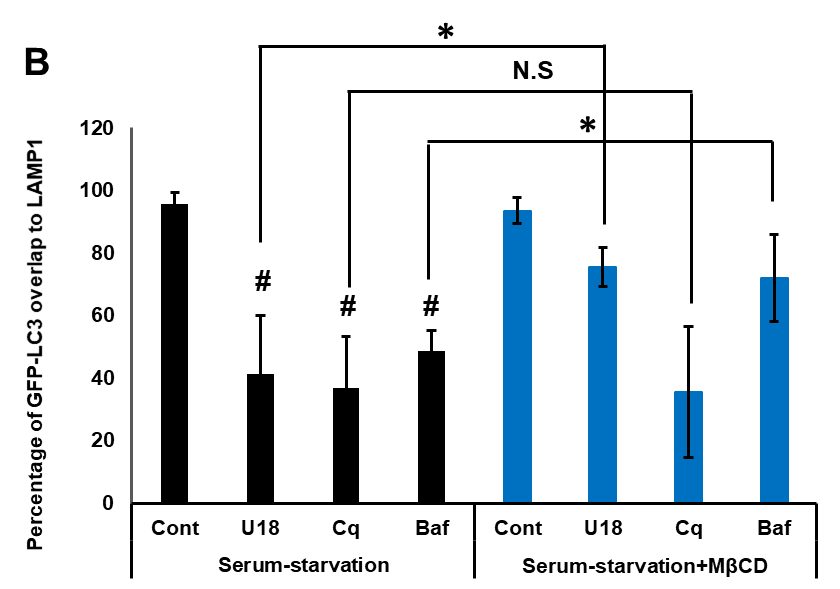
**

**Figure S2. Methyl-β-cyclodextrin enhances LAMP1 localization to GFP-LC3 positive structure in the presence of U18666A and bafilomycin A1.**

**A)** RPE1 cells were treated with U18666A (2 μg), chloroquine (5 μM) or bafilomycin A1 (25 nM) for 12 hours in a serum-starvation medium. Cells further incubated with or without MβCD (20 μM) for additional 24 hours without changing medium. GFP-LC3 transfection transfection was carried out and after 6 hours of transfection cells were fixed by 4% paraformaldehyde and immunostained with LAMP1 antibody. Scale bar, 10 μm. **(B)** Quantification of GFP-LC3 puncta overlapping with LAPM1 was quantified from 30 transfected cells by using ImageJ software. Bar graph represents mean ± SD (n=3 experiments). #P < 0.05, Student's t-test (test group were compared with control). *P < 0.05 Student's t-test (respective test groups were compared in the presence or absence of MβCD).
